# Atypical Presentation of *Aspergillus niger* Infection in the Oral Cavity as a Prediction of Invasive Pulmonary Aspergillosis in a Patient with COVID-19: Case Report and Literature Review

**DOI:** 10.3390/microorganisms10081630

**Published:** 2022-08-12

**Authors:** Mateusz Fiema, Aleksandra Wlodarczyk, Jadwiga Wojkowska-Mach, Jaroslaw Garlicki, Iwona Gregorczyk-Maga

**Affiliations:** 1 University Hospital in Krakow, 30-688 Krakow, Poland; 2 Department of Angiology, Faculty of Medicine, Jagiellonian University Medical College, 30-688 Krakow, Poland; 3 Department of Microbiology, Faculty of Medicine, Jagiellonian University Medical College, 31-121 Krakow, Poland; 4 Department of Interdisciplinary Intensive Care, Faculty of Medicine, Jagiellonian University Medical College, 30-688 Krakow, Poland; 5 Faculty of Medicine, Institute of Dentistry, Jagiellonian University Medical College, 31-155 Krakow, Poland

**Keywords:** coronavirus disease 2019, severe acute respiratory syndrome coronavirus 2, invasive pulmonary aspergillosis, *Aspergillus niger*, oral cavity, gingival pocket

## Abstract

Coinfections between severe acute respiratory syndrome coronavirus 2 (SARS-CoV-2) and other respiratory pathogens such as *Aspergillus* have become challenging, as well as being associated with high morbidity and mortality in patients with COVID-19. *Aspergillus niger* is a common environmental mold. Before the emergence of COVID-19, it was considered a very rare cause of invasive pulmonary aspergillosis (IPA), occurring mainly in immunocompromised patients. The aim of this study was to describe a very rare case of IPA caused by *A. niger* found in the oral cavity of a mechanically ventilated COVID-19 patient. *A. niger* detected in the gingival pocket was diagnosed earlier than in the bronchial lavage, and without treatment, passed into the lungs of the patient, causing serious complications. The swab from the oral cavity of mechanically ventilated COVID-19 patients can be a predictor of the subsequent severity of inflammatory lesions and the development of suspected IPA.

## 1. Introduction

Coinfections between severe acute respiratory syndrome coronavirus 2 (SARS-CoV-2) and other respiratory pathogens such as *Aspergillus* have become challenging, as well as being associated with high morbidity and mortality in patients with COVID-19 [1,2]. The SARS-CoV-2 pandemic challenges clinicians with rarely co-existing fungal infections caused by *Candida* spp., *Cryptococcus* spp., *Mucorales* spp., and *Aspergillus* spp. [3,4]. More attention should be paid to Aspergillus because it can lead to severe complications such as an invasive pulmonary aspergillosis (IPA) [5]. The most pathogenic species among *Aspergilli* is *A. fumigatus*, while twenty other species may cause infection, above all *A. flavus*, *A. terreus*, *A. nidulans*, and *A. niger* [6]. According to the data from 18 Italian ICUs, the incidence of IPA was 0.2% in 2013 [7]. During the COVID-19 pandemic, several studies and case series from Europe have reported high rates of COVID-19-associated IPA, with prevalence ranging from 20% to 35%, and an increase in the percentage of IPA in patients with COVID-19 admitted to intensive care units (ICUs) was reported (15.1%) [8].

Before the emergence of COVID-19, Aspergillus was considered a rare cause of invasive pulmonary aspergillosis (IPA), occurring mainly in immunocompromised patients (0.2%) [7].

IPA is an extremely rare condition in immunocompetent patients, but also one of the most severe forms of aspergillosis. IPA occurs especially in people whose immune systems are weakened as a result of cancer chemotherapy, bone marrow transplantation, or a disease of the immune system [9]. The symptoms of IPA are non-specific, including dry cough, shortness of breath, pleuritic chest pain, hemoptysis, thrombocytopenia, hypoxia, and acute respiratory failure [5]. Patients typically present with tachypnea, tachycardia, and hypoxia, and they are often profoundly thrombocytopenic or severely ill. The condition may deteriorate over a few days with acute respiratory failure. Diagnosing IPA remains difficult and requires a high index of suspicion. The gold standard for diagnosis is via histopathological examination and culturing of a surgical lung biopsy, but due to the patient’s severe condition, this is typically not feasible. Sputum or bronchoalveolar lavage (BAL) fungal stain and culture are commonly used methods of identification, but they are positive only in around 30% of cases [10]. Recently, noninvasive biochemical markers have been used in the diagnosis of IPA, including serum and BAL fungal cell wall antigens, such as galactomannan (GM), beta-D glucan, and aspergillus polymerase chain reaction (PCR) from BAL fluid and serum [11].

Imaging examinations involving X-ray and CT imaging of the lungs are not specific to IPA. The “halo” or “air crescent” symptom or the presence of cavities on CT lung images suggests IPA.

There are limited data available concerning the association between COVID-19 and IPA [3]. Therefore, we present a very rare clinical microbiological course of invasive pulmonary aspergillosis caused by *A. niger* found in the oral cavity of a previously healthy 64-year-old man with COVID-19 pneumonia.

## 2. Case Report

**Figure 1:**
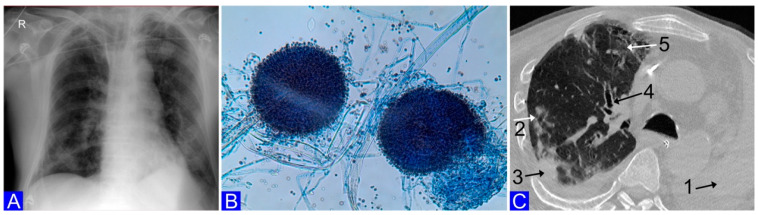
(**A**) Lung X-ray (Day 1). Bilateral pneumonia. (**B**) *Aspergillus niger*. Conidiophores of *Aspergillus niger* stained with lactophenol cotton blue (LCB), magnified 400×, provided by Zuzanna Tokarz MSc. (**C**) Lung HRCT (Day 15). Left lung atelectasis (1. right lung: suspected invasive pulmonary aspergillosis; 2. halo sign in early stage; 3. consolidation; 4. bronchiectasis; 5. ground-glass opacities).

A 64-year-old man presented with symptoms of dyspnea and general weakness. The reverse transcriptase polymerase chain reaction swab (Day 0) was positive for severe acute respiratory syndrome coronavirus 2 (SARS-CoV-2). Shading with speckled and streaked thickening, partially confluent infiltrative atelectic lesions, and partially obliterated pulmonary cavities were revealed in the chest X-ray, and pneumonia was diagnosed (Figure 1A). High inflammatory parameters were found in the laboratory tests: the white blood cell (WBC) count was 13.47 × 10^3^ cells per µL, IL-6 was 329.7 pg per mL, procalcitonin (PCT) was 24.30 ng per mL, thrombocytopenia with a platelet count of 65 × 10^3^ per µL, as well as the features of acute renal failure (creatinine, 695 µmol per L (eGFR 7); urea, 40 mmol per L). Due to the rapidly worsening symptoms of acute respiratory failure (SpO2, 50–70%; tachypnea, 40–50 breaths/min; blood pressure, 70/40 mmHg), the patient was intubated, and mechanical ventilation in assisted/controlled mode (A/C) was applied. Due to the lack of diuresis and the diagnosis of acute renal injury, renal replacement therapy was initiated by a continuous technique in the form of continuous venous–venous hemodiafiltration (CVVHDF). After intubation, material for bacteriological tests was collected (blood, urine, bronchial washes). An additional examination involved taking a smear from the gingival pocket of the teeth in the oral cavity as part of study entitled “Influence of oral hygiene on oral cavity microbiome and lower respiratory tract infections, in patients with COVID-19 ventilated mechanically”. Empirical broad-spectrum antibiotic therapy was started intravenously with meropenem (3 × 2 g) and linezolid (2 × 600 mg). In addition, dexamethasone at a dose of 8 mg/24 h was administered intravenously and continued for 14 days. Initially, the patient’s condition stabilized. On Day 1, the microbiological cultures were positive for *Neisseria meningitidis* and *Hemophilus influenzae* in the BAL and *Aspergillus niger* in the gingival pocket fluid (Figure 1B). The serum antigen tests (galactomannan, mannan, and anti-candida) were negative. On Day 15 after intubation, due to the worsening of respiratory failure, microbiological diagnostics were performed again and *A. niger* was recognized on the BAL culture. At that time, the features of an invasive fungal infection were found during computed tomography (CT) of the chest (the halo sign in the early stage, the hypodense sign, consolidation, atelectasis, bronchiectasis, and ground-glass opacities). The diagnosis of IPA was confirmed (Figure 1C). Voriconazole (2 × 400 mg p.o.) was started. Over the course of the following days, treatment with antibiotics (colistin 3 × 4 mln j. iv and cefepime 3 × 2 g iv), voriconazole, hemodialysis, and A/C ventilation was continued. Due to the prolonged need for mechanical ventilation, percutaneous tracheotomy was performed. In the following weeks of hospitalization in the ICU, the patient’s condition gradually stabilized, and his circulatory and respiratory efficiency improved. On Day 53, the mechanical ventilation was completed, and on Day 57, the patient was discharged from the ICU with passive oxygen therapy through tracheostomy.

## 3. Limitations

A limitation in this case report is that there is no evidence that the same *A. niger* strain in the oral cavity was indeed detected in BAL. However, this is very likely, as *A. niger* is not an oral mycobiote and does not routinely colonize the mucosa of mechanically ventilated patients. Genotyping techniques in microbiology, mainly bacteriology, were used, usually with a focus on outbreaks or epidemiological studies. There are limited data available concerning such methods according to *A. niger*; additionally, genotyping is costly and for this reason is not routinely performed in clinical practice [12]. Additionally, in the described case of a patient with COVID-19 in an ICU, *A. niger* genotyping was not performed, as it did not affect the therapeutic management. 

## 4. Discussion and Literature Review

A thorough literature search was performed in the PUBMED and Cochrane databases for case reports and reviews published in English-language peer-reviewed journals using MeSH terms. A search for “*Aspergillus niger*” and “COVID-19” as keywords yielded six articles, whereas the keywords “*Aspergillus niger*” and “oral cavity” yielded 24 articles. Table 1 summarizes all studies in which *A. niger* was detected in COVID-19 patients. *Aspergillus niger*, as a ubiquitous and low-virulence mold, prefers warm, moist environments. It rarely causes infections in immunocompetent people. However, emphasis should be placed on looking for aspergillosis in immunocompetent patients, especially when the infection lasts a long time and is not treated properly. The corticosteroids promote enhanced fungal growth and the invasion of *A. fumigatus* by creating a suppressive environment affecting both epithelial as well as immune cells [13]. While various human cases of *Aspergillus* infection have been described previously in COVID-19 patients [3,4,5,6], human infection caused by *Aspergillus subsp. niger* in these patients is rare. It is often described as the etiological agent of otomycosis and cutaneous infections [6,7,8]. Only five cases of *A. niger* infection have been reported previously, including four cases of invasive pulmonary aspergillosis [14,15,16,17], and a case with acute rhinosinusitis [18] (Table 1).

Laura Trovato et al. [14] described ventilator-associated pneumonia (VAP)-related pulmonary aspergillosis caused by *A. niger* in a positive COVID-19 patient. The omission of time in microbiological surveillance led to the dangerous consequence of IPA. *A. niger* proliferated and infiltrated the patient’s respiratory system, and therefore, diagnosis and administration of voriconazole were not sufficient due to the critical condition of the lung epithelium. The patient with COVID-19 pneumonia described in this case report had IPA caused by *A. niger* found in the periodontal pocket in the oral cavity. Our patient, despite sufficient microbiological supervision and the detection of *A. niger* in the gingival pocket, did not receive antifungal treatment, which resulted in IPA. Although our patient was immunocompetent, the COVID-19 infection damaged the lung epithelium and resulted in the development of IPA.

The spores of *A. niger*, inhaled with air, enter the lungs and begin to germinate in immunocompromised individuals. The mycelium that develops in the lungs begins to release toxic metabolites that inhibit the immune system, which in turn allows for further efficient growth of the mycelium. Hyphae begin to penetrate the blood vessels. This results in clots forming inside the blood vessels, thus causing local infarcts in the lungs. Tinea developing in the lungs can cause extensive damage to lung tissue, causing severe hemorrhage, which may eventually kill the patient. Infection with *A. niger* in the lungs can spread through the blood to other organs. In nearly 22% of patients with invasive aspergillosis, spread of the fungal infection from the lungs to other organs is noted [19].

In recent years, reports of *A niger* species as a serious pathogen have become more frequent. In the past two decades, *A. niger* was rarely detected in the oral cavity or nasal cavity of healthy individuals, which the major otopathogens (*Malassezia*, *Candida*) mainly colonize [20,21]. This is mainly because the lack of oral hygiene increases oral candidal colonization.

Periodontal pockets are described as specific isolated environments, characterized by appropriate biological dynamics, with two-way interactions with the oral cavity on the one hand and the circulatory system through the gingival peripheral vessels on the other. Moreover, bacterial biofilms and the presence of viruses and fungi in the periodontal pocket are of major interest to the scientific dental community. It is more and more commonly accepted that in addition to the predisposition to developing a bacterial disease such as infective endocarditis [22], viruses from the periodontal pocket can infect distant organs and thus generate focal infections. An interesting issue is the presence of fungi in the gingival pocket, which has not been sufficiently studied. There are no case reports or studies on the presence of *A. niger* in the gingival pocket and its impact on the development of a systemic infection in patients. In our patient, *A. niger* from the gingival pocket could have connected to the oral cavity via gingival crevicular fluid (GCF) and mixed with saliva, leaving the subgingival area. Another potential migration path for *A. niger* is through the periodontal capillary system and then the circulatory system. In our patient, the smear from the gingival pocket was positive for *A. niger* much earlier than for BAL, so we can assume that *A. niger* passed from the oral cavity to the lungs, causing invasive pulmonary aspergillosis. If the preemptive therapy antifungal treatment was started based on a swab from the gingival pocket, the IPA complication and lung damage would be avoided. Even though the cultures from the gingival pocket obtained from the patient were positive for the presence of *A. niger* and BAL remained negative, the patient developed IPA. The BAL was only positive 14 days after the detection of *A. niger* in the gingival pocket. Thus, we can assume that one of the most important hypotheses for the etiology of invasive pulmonary aspergillosis can be fungal biofilm formation in the periodontal pocket, which includes *A. niger*, among other pathogens. To establish the possibility of a previous chronic Aspergillus colonization in our patient, the detection of Aspergillus-specific antibodies was performed in the serum. Although BAL and the gingival pocket swab were positive, Aspergillus-specific antibodies remained negative. The negative results suggest that the patient was most likely infected in a hospital setting during intubation. According to recent data, *Aspergillus* spp. are recognized as a potential cause of VAP in immunocompetent hosts [23,24]. Colonization of the gingival pocket is an essential first step in the pathogenesis of IPA. However, the reason why *A. niger* is rare in clinical samples may simply be due to the inability to cultivate this fastidious organism. This organism is difficult to isolate using conventional culture methods, and the use of the gingival pocket swab method can increase the detection of this fungus from the respiratory tract.

The aforementioned swab from the oral cavity proved to be a predictor of the subsequent severity of inflammatory lesions and the development of IPA. The early and proper microbiological diagnosis of IPA is thus a prerequisite for its successful management. Our literature search revealed no other reports of *A. niger* found in the gingival pocket causing IPA in a patient with COVID-19. This case report is unique in that it describes the growth of *A. niger* in pure culture on conventional gingival pocket culture medium. We believe that the detection of *A. niger* in the gingival pocket in pure culture may be of clinical importance. Constant microbiological surveillance in the form of a swab from the gingival pocket will enable the early detection of *A. niger* and the initiation of preemptive treatment, preventing the development of invasive pulmonary aspergillosis.

## Figures and Tables

**Table 1:** Review of literature on the detection of *Aspergillus niger*.

Specimen	Diagnosis	Culture *n* (%)	PCR *n* (%)	References
BA *	IPA	1/1 (100%)	1/1 (100%)	Trovato, L.; Calvoa, M.; Migliorisia, G.; et al. [14]
TA *	IPA	1/1 (100%)	NP	Mirchin, R.; Czeresnia, J.M.; Orner, E.P.; et al. [15]
TA *	IPA	1/1 (100%)	NP	Pasula, S.; Chandrasekar, P. [16]
TA *	IPA	1/1 (100%)	1/1 (100%)	Singh, N.; Husain, S. [17]
FESS *	Acute rhinosinusitis	1/1 (100%)	NP	Tabarsi, P.; Sharifynia, S.; Pourabdollah, Toutkaboni, M.; et al. [18]

* BA, bronchoaspirate; TA, tracheal aspirate; FESS, functional endoscopic sinus surgery; IPA, invasive pulmonary aspergillosis; NP, not performed.

## Data Availability

Data supporting this case report are available from the corresponding author on reasonable request.
